# Mobile Technology for Healthy Aging Among Older HIV-Positive Black Men Who Have Sex with Men: Qualitative Study

**DOI:** 10.2196/11723

**Published:** 2018-12-10

**Authors:** Judy You Rong Tan, Tung T Nguyen, Alyssa Tabrisky, Robert Siedle-Khan, Anna Maria Napoles

**Affiliations:** 1 Center for AIDS Prevention Studies, Division of Prevention Science Department of Medicine University of California San Francisco San Francisco, CA United States; 2 Division of General Internal Medicine Department of Medicine University of California San Francisco San Francisco, CA United States; 3 Intramural Research Program National Institute on Minority Health and Health Disparities National Institutes of Health Bethesda, DC United States

**Keywords:** aging, HIV, black men who have sex with men, mHealth, HIV care and treatment

## Abstract

**Background:**

People living with HIV are living longer in the United States as a result of antiretroviral therapy. Black men who have sex with men (MSM) are disproportionally affected by HIV and have low rates of engagement in HIV care and treatment. Mobile technology holds promise as an intervention platform; however, little is known regarding its use among older black MSM living with HIV.

**Objective:**

The goal of this study was to explore mobile technology use and narratives of aging with HIV among older black MSM to inform mobile health intervention development.

**Methods:**

A total of 12 black MSM living with HIV, aged 50 years or older, completed in-person, semistructured interviews exploring the issues of aging, HIV care engagement, and mobile technology use. The interviews were audiotaped, transcribed, and analyzed using qualitative research methods.

**Results:**

Men appreciated having survived the AIDS epidemic, but some expressed discomfort and ambivalence toward aging. Men described various levels of engagement in HIV care and treatment; challenges included social isolation and need for support that was not focused on HIV. Almost all described using mobile technology to engage in health care, whereas some referenced important barriers and challenges to technology use.

**Conclusions:**

Findings highlighted a high level of interest toward a mobile technology–based intervention targeting older black men but also identified barriers and challenges to using mobile technology for health care engagement. Mobile technology is well incorporated into older black MSM’s lives and shows potential as an intervention platform for addressing aging issues to enhance engagement in HIV care and treatment.

## Introduction

### Background

Black gay, bisexual, and other men who have sex with men (MSM) are among the most disproportionately impacted by HIV and have some of the worst HIV care and treatment outcomes [1-3]. Due to biomedical advances such as antiretroviral therapy (ART), HIV has gone from a terminal disease to a manageable, chronic illness. A high rate of adherence (ie, >95%) to ART is required for suppression of HIV, and retention in HIV care prevents opportunistic illnesses [4]. Black MSM accounted for 26.00% (10,343) of the 39,782 new HIV diagnoses in the United States in 2016 [5]. Black MSM show the least favorable HIV care engagement outcomes relative to other racial or ethnic groups of MSM with suboptimal adherence to ART [3,6,7]. On the basis of current diagnoses rates, it is estimated that 1 in 2 black MSM will be diagnosed with HIV during their lifetime [8].

The population of individuals living with HIV is growing, aging, and experiencing a widening spectrum of diseases and conditions that compromise successful aging [9]. By 2030, as many as 73% (7503) of people living with HIV will be older than 50 years [10]. People living with HIV age earlier due to HIV infection; they exhibit multimorbidity, polypharmacy, and geriatric syndromes at a rate equivalent to those observed much later in life in HIV-uninfected persons [11-13]. Chronic inflammation and immune activation among those living with HIV are likely important in the high prevalence of aging-related conditions among individuals living with HIV [14,15]. The accelerated or premature aging has a deleterious impact on quality of life [11]. Despite an increased focus on aging with HIV and the subsequent decline in the quality of life, evidence for older black MSM living with HIV remains scant. Research is needed to inform innovative interventions that maximize functioning and quality of life as more people are living longer with HIV [16,17].

Mobile technology use among black Americans has increased with the ubiquity of mobile devices. According to a recent poll, the vast majority of black Americans own mobile devices and access the internet: 98% own a cell phone and 75% own a smartphone [18,19]. Black Americans were also more likely than whites to rely on their mobile phone for Web access [19]. Furthermore, the use of mobile technology for health, or mobile health (mHealth), is on the rise among seniors in the United States [20-22]. mHealth is a promising intervention platform, given its capabilities to mitigate traditional intervention barriers with older populations (eg, mobility) while transcending social and cultural barriers (eg, HIV stigma) [23,24].

Mobile phone–based HIV interventions have been developed for young black MSM [25,26]. However, mHealth interventions remain underdeveloped for aging populations, perhaps due to misconceptions about user capability despite evidence to the contrary [27-29]. A recent study showed that older black men indicated high willingness to participate in mHealth interventions, despite never having participated in them [30]. Furthermore, older black men were willing to participate in mHealth if they were offered more information about the topic or if they trusted that the research targeted and benefitted racial/ethnic minorities [30]. Another study found that the association between age and technology use was mediated by the effect of computer-related anxiety, self-efficacy, and confidence, suggesting that interventions that address these psychological factors can improve use of mHealth among older adults [31].

### Objectives

To our knowledge, there are no technology-based interventions specifically developed for improving HIV care and treatment among older black MSM [25,32,33,34]. Research must first characterize the needs and preferences of older black MSM living with HIV who are increasingly in need of viable innovations adaptable to various social and behavioral factors that promote healthy aging. Formative research can help identify critical factors that could affect intervention feasibility, acceptability, and retention/attrition. Therefore, the goals of this study are to explore using qualitative research methods, to assess the issues involved in aging with HIV, and to understand the unique needs and preferences among black MSM to inform mHealth intervention development for enhancing HIV treatment outcomes.

## Methods

### Participants

Participants were recruited using purposive sampling to participate in a one-time in-person interview to explore the role of mobile technology in care engagement among older black MSM living with HIV. During a 3-month period, recruiters distributed study materials containing a dedicated study phone number at AIDS service and community-based organizations serving black MSM in (blinded for review) and social venues (eg, local bars and cafes). Potential participants were screened by trained research staff on the phone for eligibility. Individuals were eligible if they identified as black or African-American and as cisgender men at the time of the study, were currently living with HIV, owned a personal mobile telephone device, and were aged 50 years or older. We chose this age threshold because overwhelming evidence demonstrates that aging syndromes (eg, frailty and HIV-associated neurocognitive disorders) occur earlier (ie, by age 50 years) in people living with HIV. HIV status was verified by a letter of diagnosis or by a labeled pill bottle of their HIV medicine.

Eligible participants were interviewed in a private room located at a community-based research site accessible by public transportation. An interview guide was used to explore overlaps among the topics of aging, HIV care and treatment, and mobile technology. Sample interview questions include “In a typical day, what do you use your mobile phone to do,” “What does aging mean to you,” “What does living with HIV mean to you,” and “How do you use your mobile phone in your health care?” Each interview lasted approximately 1.5 hours and was audiotaped and transcribed for analysis. All study procedures and materials were approved by the lead author’s institutional human research review board.

### Approach

The main goal of the study was to explore the intersection of aging, living with HIV, and mobile technology to inform mHealth intervention development for improving HIV care engagement among older black men living with HIV. Qualitative analysis entailed reading all transcripts, highlighting sections of texts to derive themes based on narratives of aging and technology use in the context of HIV, and care engagement. Next, each transcript was summarized, and themes and subthemes emerged under the domains of mobile technology use in general and for HIV care engagement and aging with HIV as a black man. The themes and subthemes were reviewed by the senior author and coauthors (with supporting excerpts from the text) and revised as needed. Thereafter, the first author coded and created analytic memos based on emergent findings.

## Results

### Overview

A total of 12 black cisgender men (mean 57.7 [SD 6.5] years) living with HIV participated in one-on-one, semistructured interviews. Of these, 9 men reported receiving or applying for social security disability benefits; 3 men had full-time employment. The annual household income ranged from less than US $10,000 to $55,000. Moreover, 6 men (50%, 6/12) reported having a primary relationship partner. All men reported taking antiretroviral medications currently. The length of time on ART ranged from 3 to over 22 years.

We identified 4 major themes (with subthemes): (1) use of mobile technology, (2) use of mobile technology in HIV care, (3) the meanings associated with aging with HIV, and (4) mHealth design implications. In the following sections, we first present how men described using mobile phone technology, particularly with respect to HIV care engagement, and the barriers and challenges to its use. We present how men engaged in HIV care and treatment, including barriers and challenges the men referenced. Next, we present findings of what it means to age with HIV. Finally, we present mHealth design needs and considerations.

### Use of Mobile Technology

Mobile technology literacy, reliance, and habits ranged widely. Some participants described being very technologically savvy and reliant on their phone. Some had at least two mobile devices. For example, a 50-year-old participant reported having 2 mobile phones, 1 for business purposes and the other to listen to music. Another participant also reported owning 2 mobile phones, an iPhone and an Android (ie, the *Obama phone* for eligible Medicaid recipients in California); he preferred the iPhone but has the other phone because he could not always pay for the service on the iPhone. He explained that he mostly used texting as a form of communication but used an iPad for email. He has other apps on his phone for accessing the news (eg, Huffington Post and BBC), social media (eg, YouTube), and dating sites (eg, Grindr). Other than calling and texting, the most common use of the mobile phone was to listen to music. One man explained that it is easy to use music apps relative to other features:

I really like my music. It’s really easy. I use Spotify. And it’s really easy to get along and navigate through the different prompts. I hate texting. I’m not that fast of a typer. I don’t even like to use emails. [I use my phone for] calls and music and semi-texting.

### Challenges to Mobile Technology Use

Participants perceived the following barriers or challenges to using mobile technology: having low technology literacy, feeling uncomfortable using mobile technology, having limited access to data (affordability of data plans), and having privacy concerns. Some lamented being unfamiliar with features and described frustrating experiences, including the use of social media apps. One man referred to feeling overwhelmed when using Facebook:

With my [previous] Android, I had contact with people on Facebook. I was overwhelmed. I couldn’t go on it anymore. Have not even adapted my [current] phone to do a Facebook. I’m overwhelmed by the number of conversations. Well, no. I don’t worry about some-my sister. She Facebooks everything and I don’t need to know everything. I don’t want to know everything.

Of the 12 men, 6 men between the ages of 50 and 69 reported using social media apps. Of these 6 men, 2 referred to using both Facebook and Instagram or Twitter and Instagram. Moreover, 1 participant mentioned that he was “not very tech savvy” and used his phone primarily for texting, calling, and listening to music:

It can be a headache, my phone. I’m not all that savvy when it comes to technology and what not. But I use it for the basic necessities. I’m not glued to my phone daily.

Despite frustrations, many participants described that they “cannot live without the phone.” A 66-year-old man with an iPhone described still learning how to use all of the features and occasional challenges. However, he described his phone as indispensable to everyday life. He used mobile apps to stream music, email, and keep track of his appointments. He also owned a tablet at home that he used to surf the Web.

Frustration with using mobile technology was almost always related with unfamiliarity with various features and navigating certain *bells and whistles*. Other challenges to using mobile technology included the hassle of having to register a user name and password. For example:

Sometime it’s complicated to find different things, how to work it, what you have to do to this, downloads and that kind of stuff. I’m not good with that kind of stuff. I’m not patient with that kind of stuff. I hate like going to sites where you have to register and all that kind of crap. If I have to do all that, forget it.

### Use of Mobile Technology in HIV Care

Regarding use of technology related to health care, most participants reported using mobile apps in addition to making phone calls to engage in health care. In addition, 1 man indicated that he kept track of his medical appointments by setting reminders on his mobile phone devices. He explained that he used a pharmacy app to keep track of his medication refills and will receive a text alert when his medications are ready for pickup. He mentioned that he receives his lab test results via email and discusses them with his doctor. Some men used their mobile phone to Google information on symptoms or to stay on top of medical news such as the newest ART. One participant expressed:

Well, first of all, I’m an avid news fanatic. If anything comes up, and it catches my eye, I’ll check into it. I mean, the progress with cure stuff is coming. But if I see something, I’ll bring it up to a physician and say, what do you think about this, or what do you think about that?

It is important to note that although many participants were reliant on and somewhat proficient with advanced mobile technology features, there were a few men who used the bare minimum. A few men were unable to access apps and other features because they did not own a smartphone, highlighting barriers to mobile technology for health care.

Despite a wide range in proficiency, almost all described mobile technology use in HIV care engagement. A 68-year-old man described not being too savvy and having an outdated phone but also showed some advanced use of technology for engaging in health care: he explained that he accessed all of his labs and physicians via their websites. Another man described his technology use to be very minimal; he nevertheless described using the app from his pharmacy, which sends him a reminder for prescription refills. Another man described using various apps, such as housing apps to find housing and a music app. He stays organized by using the calendar on his phone to set reminders. A 64-year-old man described using his mobile phone to research medications, conduct banking, and find social support group meeting times. He referred to using several apps, including the calendar on his phone, and social media (eg, WhatsApp and Facebook).

### Aging With HIV

#### Loss of Opportunity

For several men, living through the HIV epidemic meant a loss of real opportunity and time to think and plan ahead. For the most part, these men accepted the widely held belief in the early days of the epidemic that they would never have to face getting older because they would die of AIDS first. For some, expecting to die young precluded putting hopes on one’s future, as a 57-year-old man expressed:

Yeah, I don’t think that far ahead, though. I can’t. I have to think day by day and what I’m going to do today or tomorrow or next week. I don’t like to think too far ahead. But I hope I’ll be in a good place. I hope I’ll be in a good place mentally. I hope my health still be holding up.

For the generation of men who expected to die of AIDS before ART, having lived through the epidemic only to now face the inevitability of aging was bittersweet. Some expressed regret and disappointment at the years lost. For example, a 51-year-old man described his disappointment in realizing that he would have lived differently if he had known HIV would not be a death sentence:

I didn’t think I would be alive at this point. I expect to get older now versus I didn’t in the past. It’s amazing to me, so it’s great to age, to be alive when so many people have passed away. I was mad at the doctor forever, because he told me I wouldn’t live to be 30. Oh, I wasted so much time. I could’ve finished school and everything. I started like spending my retirement money and going to Europe. [But I was] not getting sick. And ten years go by, it’s like, oh, yeah, you’re a long-term non-progressor. Ooooh, that doctor.

#### Avoidance

Some men held the idea that aging meant becoming infirm, being an invalid, and growing surly and mean over time. This was in contrast with how they viewed themselves currently, representing a separation between aging as a negative concept and their self-perception. One participant described aging as “gray hair, disappointment at what you have not achieved, going forward with what you have, and trying to make the best of the rest of your life.” Another 50-year-old participant expressed his dismay when someone younger referred to him as *Pops*:

And a kid called me Pops. He got up. I was on the bus, and he said, here, “Pops, here’s a seat for you.” I said, now I look old? I don’t want to look all old, and I just don’t want to be old and mean like my parents did when they got older.

The concept of aging was often synonymous with dying. A 56-year-old man explained avoiding thinking about aging and the impending *move toward death*:

It’s the end-of-life thing is what I get reminded of as getting older. And I don’t like to think about that, even though I’m living it and moving toward that. And I guess that’s my way of not liking it so much. Because I don’t want to progress and move forward towards death and dying. I just don’t. I just-I don’t look forward to it. I know that it’s inevitably going to happen. But I try to erase some of that in my thinking...

Some men admitted the increasing number of challenges in getting older and facing multimorbidity, including HIV. A 68-year-old cancer survivor explained that he was “not a friend of aging”:

It just means the body wants to fall apart. And how am I dealing with it? Well, I have struggles. I have good days and bad days with aging. I’ve had two health issues that [have had to do with me] aging. So I have my good days and my bad days. I’m not a friend of aging so much.

Some participants avoided thinking about aging and HIV. One man reported that he did not like to think about aging and the changes it inevitably brings. He became sad when referring to his HIV status. When asked if he attended support groups, he explained:

I don’t really go to any support groups...because I don’t like really talking about it. I very seldom talk about my HIV problem. I done came to terms in myself that I have it, and I have to take care of myself. But I don’t really like talking about it. It depresses me.

He referred to receiving drug counseling, which has helped him with his mental state. He mentioned that it was difficult for him to find a partner because he does not like talking about his HIV status:

I don’t want nothing to remind me that I have HIV. I know I have it, but I don’t want to be talking about it. I’m going to take my medicine, and that’d be it. I don’t even like to think about it, which is probably not a good thing, but I haven’t been stressed over it. So I don’t really trip off of it. The only thing I regret is - the only thing that hurts me about it is like finding a partner. Simple fact that I don’t like talking about it, and I would have to tell them.

#### Acceptance

In contrast, some men appeared to accept the idea of getting older and the inevitability of death. Many were thankful that they were able to manage HIV as a chronic disease, emphasizing the pointlessness in dwelling on the negative. A 63-year old described viewing aging with HIV as just part of life:

I have a good positive attitude about it. I don’t think negative about it. It’s nothing you can do about aging. It’s just life. You just have to deal with it and make the best of it you can. And when you don’t dwell on it. Sometime you may get a little bit depressed, but I kind of get out of that depression because I think stuff could be worse. And I’m lucky to get around and do what I have to do... You got to keep yourself motivated, keep yourself going. You just got to keep moving.

Another man described how he maintains perspective on living with a chronic but manageable disease like HIV in terms of how things could be so much worse:

There’s a lot of other things could be going on if I lived in a different part of the world. What is it like living in a poor neighborhood in Libya? What is it like in Syria for kids right now? Some people would do anything to be in my position or live on this planet. So I don’t take it lightly, but I appreciate that it could be so much worse.

Some men understood aging in the context of surviving or *defying* HIV and expressed pride in having survived HIV. A 68-year-old man expressed appreciation for being alive when so many of his peers died during the AIDS epidemic:

Well, I was diagnosed in ’85, so I’ve pretty much got the kinks out of the HIV craziness because I-many years of craziness around HIV, many years. So now I just take my medication. Actually, my relationship with HIV is, I’m really glad. Last Tuesday was my 68th birthday, so I’m really glad to be alive.

In addition, 1 man cited having a *fighter* attitude and admitted that he still looked forward to living a healthy and full life:

Because I ain’t ready to die. I feel I still got things to do and people to hang out with. So, yeah, I’m just not ready... I looked at so many people died because they didn’t [take care of their health]. I don’t want to go out like that. I don’t want to be all messed up because I didn’t take care of myself. I just want to -I want to live. That’s what motivates me. Because if you don’t, you die. I ain’t trying to go out like that. I’m a fighter, literally.

Some appreciated the opportunity to see old age. One man recounted an early memory of his aging mother describing to him what it was like to grow old and now appreciating being able to reflect on his own experience in aging:

I’m quite comfortable with being 50. I’m surprised I made it this far. And I remember when my mother was 50, had turned 50. And I was thinking about all the things that she was trying to tell me that was going to happen when you turn 50. I just don’t find a lot of pleasure in things I used to when I was 40. I look at -some of them I think are stupid now. And I’m set in a certain way. I’m comfortable with it.

When asked how he feels about aging, one man described finding *unmerited favor* in living into old age with HIV:

Participant: It means finding grace in the midst of-let’s say the physical, emotional, and spiritual condition, because I realize that there are things that have changed from my youth, and to make sure that I continue to focus on not what is leaving, but what I can still do.

Interviewer: What do you mean by grace

Participant: I mean unmerited favor. I mean the opportunities to live through wisdom, to be able to tap into a power source greater than the circumstances and situations, having faith in a process that relieves me from anxiety and fear.

Some participants expressed that attitude was a major factor in aging. One man considered himself a healthy individual despite living with HIV. He mentioned that having a good attitude was the key to staying healthy:

It’s not what’s thrown at you, but how you deal with it, and that all resorts back to state of mind-just to be able to not let those things affect you to the point where you’re stressing, because that’s a silent killer. Yeah, I think the longevity issue is how you deal with a situation that you’re dealt.

Spirituality and religion appeared to play a role in some men’s viewpoints on aging. One man used to attend a gay-friendly church to *take bits and pieces* of religion. He wanted to make the most of what the time he has left in life, referring to the universality of aging:

It’s getting better, understanding life a little better, knowing that one day I’m not going to be here. So do the best I can with what I got and face it. I’m still here. I’ve been positive since 1990. As long as I could walk and talk and get around to the best of my ability, I’m fine. I’m going to die. All of us are going to die one day.

### Mobile Health Design Implications

#### Willingness and Acceptability

There was a moderate level of acceptability of a mobile technology–based tool, such as an app, that would be designed for older men living with HIV to support care engagement. One participant appreciated the mHealth research focus on older men of color living with HIV (“I think what you guys are doing is an asset. And I think [an app for us] would be great. It would be nice to see that. I definitely would support that.”). Many participants were interested in using a mobile app to organize care engagement and access information in 1 central place. However, interest in using an app was qualified by an emphasis that the app would have to be easy to use:

[I would be interested] if it’s user-friendly. I get frustrated with difficult prompts, and I’m not that - I don’t have the patience for that. But if it’s user-friendly, yes, I would be interested in it.

Given memory loss, most men saw the value in using an app as an additional tool in helping to remember things like medical appointments or social engagements:

I would be interested [in using an] app might have a weekly or monthly reminder of something, you know. Because truth be told, to be reminded of something, I think it’s very important and very helpful to a lot of us that are out there. Because, like, when you got your medicine and all this other stuff on your mind, the little reminders [would help]. For instance, I have my yearly housing thing coming up where, they sent me a thing in the mail which I got up on my bulletin board, but...you get a lot of mail sometimes, you know. I’m 62 and my brain’s kind of working still, but I can sense [that] the older I get—I could appreciate prompts and reminders with a lot of things.

#### Connecting With Similar Others

Overall, 4 of the participants reported belonging to a social support group of older men living with HIV. Acceptance of older age appeared to be associated with being connected with similar others. Furthermore, 1 man explained that socializing with similar others helped him learn about or understand some of his own aging experiences:

I’m still trying to identify with [getting older]. I mean, that’s why I go to my groups. Then we all speak about it. Then I learn from there. My body is aging a little bit because I’m starting to feel it...we have a weekly Wednesday group which is like a support group…it’s the little social things we do that really make me feel good and the camaraderie, you know.

A few men mentioned the need for the existence of such social networks for older men living with HIV. One participant mentioned it would be helpful for a mobile tool such as an app to connect men with each other and remind them to attend groups:

Just something to ring a bell once [that] this group is going on at a certain time. And maybe anything that that group is doing...[like] get little notes about certain functions that group is doing. And because a lot of us are getting older it’s hard to get sociable, but...I think if they knew things were going on [and] got reminded, they would go out. But yeah, something like [a way to connect socially and get reminders] that [is] simple and steady, I could appreciate it.

Other men who did not belong to a formal social gathering expressed interest in attending support groups devoted to older folks living with HIV for the purpose of learning from others and contextualizing their experiences about aging with HIV. For example, 1 man expressed interest in app features that would provide reminders and connect him to others living with HIV from whom he could learn their experiences with HIV care. He mentioned that although he did not see a need for reminders to take his medications, he saw the value of getting reminders for his medical appointments:

[I want to know] how are they taking their medication? Are they going through anything, like side effects? Because I don’t forget to take my medicine. I think that probably [I] might need something like a reminder for a doctor’s appointment.

Health concerns among older men living with HIV were mostly unrelated to HIV but rather on the lack of guidance and focus on healthy aging. Men expressed a need for targeted messages that focus on their resilience in having survived the epidemic and on friends and family. Men who had been living with and managing their HIV for many years indicated that social support should not focus solely on HIV but rather on overall health and addressing barriers to healthy aging. These men emphasized that social spaces for older men living with HIV should have *a positive spin on golden years* because they felt that the focus these days was all on being and staying undetectable. Social groups that focus on healthy aging (physical and mental health) and the golden years may be a way to attract men who would otherwise be averse to interventions that focus solely on HIV:

I very seldom talk about my HIV problem. I done came to terms in myself that I have it, and I have to take care of myself. I had been going to drug counseling, actually, that was helping a lot as far as my mental state.

Several men who were less engaged in care and treatment reported being somewhat isolated, underscoring a critical gap that mobile tools may fill by facilitating access to necessary resources. When asked whether he would be interested in using app features to connect with others, one man explained:

Simple fact that I don’t like talking about [my HIV status]. So basically I’ll be by myself. That’s one reason why I be by myself a lot... I haven’t been with anybody because I’m too scared of rejection. So yeah. And I probably would [be interested]. I probably would. Because sometimes I just be needing somebody to talk to about various issues. So that may work. I’m just a quiet person, and I don’t really like to [talk] on the phone.

#### Support for Self-Management

Participants voiced preferences for educational content on communication and self-management. Some men mentioned wanting to learn skills to manage stress and anger. Furthermore, 1 man explained the need for having tools, such as a daily planner and reminders, to support better time management:

For me, time management is important. We have so many things to do within a day that we don’t get done. So if we could design an app that would take you from the time you get up to the time you lay down, things you need to do, a to-do list, contacts, implementing those, check off, and just feeling like you got through the day and you got some things done, I think that would be awesome. That would be also helpful to know that I got an alarm that will wake me up, maybe a scheduled time every morning, if I have certain things I do, a regimen, get up, walk the dog, have my coffee, breakfast, phone calls. Then I’ll make my way to work. If I got appointment reminders, just little stuff that would kind of be in your ear every day, that would be great. Sometimes I do forget [to take my medicine]. Right now I’m switching meds, so it’s important for me not to miss a dose. So, yeah, reminders would be great.

#### Concerns About Confidentiality

A majority of the participants mentioned using passcodes on their mobile devices. A few men appeared reticent to use an app that would contain personal health data due to concerns about confidentiality. Furthermore, 1 man explained his concern about a loss of confidentiality trumping his desire to access his medical information in 1 central place on his mobile device:

I’d be worried about why all that information’s in one place. Why would I want to have all my information linked where if I make a mistake, all of it’ll be out there? With all this hacking and nonsense, I don’t know if I would want to use it or if it would even be necessary, because right now it’s not.

## Discussion

### Principal Findings

This study explored mobile technology use and narratives of aging with HIV among older black MSM to inform mHealth intervention development. The findings centered on 4 themes: (1) mobile technology use in general and (2) in HIV care, (3) aging with HIV, and (4) mHealth design implications. Conceptualizations of aging with HIV were often situated within narratives of the early AIDS epidemic and what it means to be a long-term survivor when many others have perished from it. Many considered themselves to be fortunate to live long enough to age, with several of these men accepting the inevitability and universality of aging. By contrast, others viewed aging negatively as becoming infirm and unable to live life to the fullest extent. Findings underscored a need for promoting models of healthy aging relevant to black men living with HIV, given findings that conceptions of aging are culture- and context-dependent [35]. Many men aging and living with HIV may lack visible and positive role models, perhaps due to the death of peers and social isolation exacerbated by social factors related to HIV stigma, aging, or both [36-38].

Findings also showed a wide range in the level of comfort and skill in using mobile technology. Many older black men relied heavily on their mobile phones to access entertainment such as music. The findings were encouraging of leveraging mobile technology as an HIV intervention platform: older black men relied on their mobile phones specifically in their HIV care, such as ordering their medications via drugstore apps or calling their providers to schedule appointments. Several reported using self-management apps (eg, calendar) and functionality (eg, reminders) to organize HIV care and treatment. Finally, we found evidence of acceptability of an mHealth intervention in the form of an app for enhancing HIV care and treatment among older black MSM.

These findings show where needs of older black MSM are similar to or different from those of young black MSM [39]. Similar to young black MSM, older black men considered confidentiality and privacy in mobile technology to be paramount [39]. Younger black men preferred a holistic approach to health in an mHealth intervention rather than one solely focused on HIV [39]. Similarly, older black men desired access to information on successful aging and health concerns unrelated to HIV. Regardless of age, men cited self-management as an important reason for using mobile technology. In contrast, although younger men relied heavily on apps [39], our findings with older men showed variability in the level of reliance on and comfort with using certain mobile phone features. Although some younger black men wanted to seek experienced *sponsors* for guidance and support [39], older black men were more varied on their desire for Web-based social connections. Future mHealth research should explore how a mentorship model facilitated by mHealth may be appropriate for connecting younger and older black MSM toward achieving better health.

### Implications for Intervention

Our findings point to specific content areas that an mHealth HIV intervention should address with older black MSM. Interventions that promote positive conceptions of aging with HIV may be particularly impactful. Promoting ideas about resiliency and the *golden years* may help men who otherwise avoid the topic incorporate holistic and dynamic models of aging into their self-concepts. Social isolation among the elderly is common, but among those living with HIV who are also sexual and/or gender minorities, social ties may be even more tenuous [40,41]. Areas for focus include information on healthy aging, connecting socially to similar others, and self-help aspects such as managing psychosocial distress. Men who wish to connect in person with others for social support may already belong to formal groups; those who are more socially isolated may want the option of connecting remotely to potential social groups at first. Thus, access to existing social support networks should be remote as well as in person [18]. Since many older men had been engaged in care and treatment for some time, content around HIV may be deemed irrelevant. In addition, some men expressed being tired of HIV prevention and health intervention messaging solely on HIV. mHealth interventions should focus on helping users define and achieve goals of aging along with promoting HIV care engagement.

Findings also guide the design of mHealth features. App features should be easy to navigate and focus on being operable right off the shelf, minimizing user burdens and cognitive loads. For example, usernames could be set to mobile telephone numbers rather than a unique username that may be hard for many to remember. The number of features should also be minimal, with a focus on simplicity and enhancing user experience. The tool should include reminding and camera functions the users are most likely to be familiar operating. Additional features may include day planners. Incorporating mobile phone capabilities such as music, social media, and video streaming may be a viable way to engage older black men [42].

### Limitations

The findings are limited to a small sample size due to the pilot nature of the study. This study did not extensively explore how challenges to using mobile technology might be associated with specific aging-related cognitive, physical, perception, and motivation barriers to mobile technology [21]. Given the goal of the study, the small sample size, and sampling method, findings cannot be generalized. Eligibility for the study participation included owning a mobile phone, although ownership of a smartphone was not a criterion. A small minority of participants did not own a smartphone, even as smartphone devices are affordable through safety net, state-subsidized programs. Even as rates of adoption increase among older racial/ethnic and sexual and gender minorities, researchers are cautioned to consider existing constraints on mobile technology access.

### Conclusions

Adoption of digital technology among older black MSM living with HIV is likely influenced by a multitude of issues [22]. This study adds to the scant literature specific to older black MSM living with HIV, a population showing health disparities and for whom no mHealth interventions exist [42]. Mobile technology for promoting healthy aging with HIV among older black MSM appears acceptable and is likely to be feasible based on extant digital technology for advancing black men’s health [18]. mHealth interventions for older black men living with HIV can educate and promote healthy concepts of aging with HIV and support self-management skills and behavior around HIV care, such as adhering to ART [18]. It is paramount that future research to develop mHealth interventions work with black MSM to explore culturally relevant mHealth strategies rather than apply a *one-size-fits-all* approach.

## Abbreviations

ART: antiretroviral therapy
mHealth: mobile health
MSM: men who have sex with men
